# Impact of Microplastics on Ciprofloxacin Adsorption Dynamics and Mechanisms in Soil

**DOI:** 10.3390/toxics13040294

**Published:** 2025-04-11

**Authors:** Qian Xu, Hanbing Li, Sumei Li, Ziyi Li, Sha Chen, Yixuan Liang, Yuyang Li, Jianan Li, Mengxin Yuan

**Affiliations:** 1 Department of Environmental Science, College of Environmental Science and Engineering, Beijing University of Technology, Beijing 100124, China; qianxu@emails.bjut.edu.cn (Q.X.); hanbingli@bjut.edu.cn (H.L.); liziyi0729@163.com (Z.L.); chensha@bjut.edu.cn (S.C.); liangyixuan_bjut@163.com (Y.L.); yyangli112@163.com (Y.L.); 13892290045@163.com (J.L.); 15698997803@163.com (M.Y.); 2 Key Laboratory of Beijing on Regional Air Pollution Control, Beijing University of Technology, Beijing 100124, China; 3 Meteorological Bureau of Haidian District, Beijing 100080, China

**Keywords:** microplastics, soil, adsorption, ciprofloxacin, mechanism

## Abstract

The co-occurrence of microplastics (MPs) and antibiotics as emerging contaminants demonstrates significant ecological perturbations in soil matrices. Of particular scientific interest is the potential for MPs to mediate the environmental fate and transport dynamics of co-existing antibiotics. This study investigated MP-mediated ciprofloxacin (CIP) adsorption in lateritic soils. Batch experiments with polyethylene (PE), polypropylene (PP), and poly (ethylene-terephthalate) (PET) revealed soil components dominated CIP retention, while 10% (*w*/*w*) MPs reduced soil adsorption capacity by ≥10.8%, with inhibition intensity following PET > PE > PP. Adsorption thermodynamics exhibited significant pH dependence, achieving maximum sorption efficiency at pH 5.0 (± 0.2), which was approximately 83%. Competitive adsorption analysis demonstrated inverse proportionality between ionic strength and CIP retention, with trivalent cations exhibiting superior competitive displacement capacity compared to mono- and divalent counterparts. Isothermal modeling revealed multilayer adsorption mechanisms governed by hybrid chemisorption/physisorption processes in both soil and MP substrates. Spectroscopic characterization suggested differential adsorption pathways: MP-CIP interactions were primarily mediated through hydrophobic partitioning and π-π electron coupling, while soil–MP composite systems exhibited dominant cation exchange capacity and surface complexation mechanisms. Notably, electrostatic attraction/repulsion forces modulated adsorption efficiency across all experimental conditions, particularly under varying pH regimes. This work advances understanding of co-contaminant dynamics in soil ecosystems, informing risk assessment frameworks.

## 1. Introduction

Antibiotics constitute a class of pharmacologically active compounds extensively employed in clinical therapeutics, livestock production, and aquaculture operations for disease mitigation and growth promotion [1,2]. Following metabolic processes in humans and livestock, approximately 59% of administered antibiotics are excreted into environmental matrices either in parent forms or as biotransformation derivatives [3]. Concurrently, microplastics (MPs), defined as synthetic polymer particulates with dimensions < 5 mm [4], have emerged as pervasive contaminants in terrestrial ecosystems. Their nanoscale dimensions and elevated specific surface area (SSA) facilitate the sorption of hydrophobic organic compounds and metallic contaminants, thereby serving as transport vectors that modify pollutant distribution patterns and induce complex ecotoxicological effects [5,6,7,8,9,10]. While substantial research efforts have been devoted to aquatic systems regarding MP–pollutant interactions [11,12], terrestrial environments exhibit comparable MP contamination levels [13,14,15,16,17]. Notably, MPs undergo progressive weathering in soil matrices, potentially releasing sequestered contaminants through degradation processes and establishing persistent pollution cycles [18]. Systematic investigation of MP–antibiotic interfacial interactions in pedological systems provides critical theoretical foundations for (1) ecological risk assessment of MP contamination and (2) understanding multipollutant synergistic effects in terrestrial environments.

Currently, the adsorption behavior of antibiotics in aquatic and soil environments has attracted widespread attention. However, limited research exists on the interactions between microplastics (MPs) and antibiotics in soil systems, leaving our understanding of their combined environmental impacts incomplete [19,20]. Recent findings indicate that MPs can suppress antibiotic degradation and enhance the mobility of certain antibiotics, such as oxytetracycline [15]. Previous studies have investigated the adsorption and degradation of various antibiotics in agricultural soils, including tetracycline, sulfamethoxine, norfloxacin, erythromycin, and chloramphenicol, with tetracycline showing the highest adsorption capacity [21]. Additionally, soils rich in organic matter and aluminum/iron oxides exhibit stronger adsorption of tetracycline antibiotics [22]. Several factors influence antibiotic adsorption in soil, such as increased pH and ionic strength, both of which reduce tetracycline adsorption [23]. Furthermore, incorporating 10% MPs into soil decreases its ability to adsorb oxytetracycline [24]. Despite these advances, significant gaps remain in understanding antibiotic adsorption dynamics within soil–MP composite systems.

In summary, most of the existing studies focus on the adsorption behavior of antibiotics on microplastics, as well as the adsorption and migration of antibiotics in soil. There are few studies on the adsorption and migration of antibiotics in complex soil environments where microplastics exist, so this study compares the adsorption behavior of antibiotics on MPs, soil, and soil–MPs complexes to provide a scientific foundation for understanding the migration and transformation dynamics of antibiotics in soil environments contaminated with MPs. Ciprofloxacin (CIP), a member of the fluoroquinolone antibiotics (FQs), is resistant to hydrolysis and volatilization, accumulating in soil with strong sorption affinity but limited mobility [25]. Due to its inability to be fully metabolized in the body, most of it is discharged into aquatic systems via feces, urine, and other pathways [26]. In this study, CIP was selected as a model compound to investigate the adsorption behavior and mechanisms of antibiotics in soil systems containing MPs. Red soil (Typic Haplustults), which constitutes a significant portion of Chinese soils, was chosen as the test medium. To examine the influence of MPs on CIP adsorption, various types and concentrations of MPs were introduced into the soil. Additionally, experiments were conducted under different pH levels, ionic strengths, and MP types to elucidate their effects on CIP adsorption in soil–MP systems.

## 2. Materials and Methods

### 2.1. Materials

The red soil was purchased from the Institute of Geophysical and Geochemical Investigation, Chinese Academy of Geological Sciences, the basic physical and chemical properties of which are shown in Table S1. Ciprofloxacin, NaOH, HCl, KCl, CaCl_2_, and AlCl_3_ were purchased from Maclin Biochemical Technology (Shanghai, China), which were analytical grade or higher. Ultrapure water (MQ) was obtained from a Milli-Q water purification system (SD-UPT-10, Sichuang Youpu, China). Polyethylene (PE), polypropylene (PP), and poly (ethylene-terephthalate) (PET) were purchased as powders from Maclin Biochemical Technology (Shanghai, China). For any kind of MPs, more than 90% of polymers fell into the 25–100 μm size classes.

### 2.2. Adsorption Experiments

(1) Adsorption isotherm experiments

The adsorption isotherm experiments were performed in 100 mL amber bottles. For MP samples, 40 mg of MPs were added to each bottle along with 10 mL of antibiotic solutions (4, 6, 8, 10, 12, 15, 20, and 25 mg/L). The mixtures were then incubated in a thermostatic shaker at 25 °C and 165 rpm under constant light. Sampling intervals were determined based on kinetic trends. After shaking, the supernatant was filtered through a 0.22 μm PTFE membrane, and antibiotic concentrations were quantified using UV-Vis spectrophotometry. All experiments were conducted in triplicate, with results reported as mean values.

(2) Soil adsorption isotherm experiments

Soil adsorption studies utilized four sample types: pure soil (100 mg) and soil amended with 1% PET, PP, or PE (100 mg each). Each sample was placed in a 100 mL amber bottle, mixed with 8 mg/L antibiotic solution, and shaken at 100 rpm (25 °C) for 24 h using a thermostatic oscillator (THZ-D, Jiangsu Jiamei, China). Aliquots were collected at specified intervals (5 min, 10 min, 30 min, 1 h, 1.5 h, 2 h, 4 h, 6 h, 12 h, and 24 h), filtered through 0.22 μm PTFE membranes, and analyzed as described above. Triplicate measurements were performed to ensure reproducibility.

The effects of pH, MP dosage, ionic strength, and ion type on CIP adsorption were investigated. The pH effect was examined at room temperature by adjusting the solution pH from 3 to 9 using 0.1 mol/L HCl or NaOH. To study the impact of MP dosage on CIP adsorption in soil, experiments were conducted with MPs set at 0%, 1%, 5%, and 10%. Higher MP dosage simulated severe pollution scenarios like extensive agricultural film residues or landfills. Ionic strength effects were explored through varying NaCl concentrations (0, 0.001, 0.01, 0.1 mol/L). Ion-type effects were analyzed using KCl, CaCl_2,_ and AlCl_3_ solutions at 0.01 mol/L.

### 2.3. Analysis Methods

(1) Characterization methods

The remaining concentration of CIP in the solution was measured by a UV-visible spectrophotometer (TU-1900, Beijing Puxi, China) at a wavelength of 270 nm.

The polymers’ microscopic morphological characteristics were analyzed by a Scanning Electron Microscope (SEM) (ZEISS Gemini 300, ZEISS, Oberkochen, Germany). The charge profiles of MPs were analyzed by a zeta potential analyzer (ZetaPALS, Brookhaven, NY, USA). The crystalline compositions of MPs were measured using an X-ray diffractometer (XRD) (SmartLab SE, Rigaku, Tokyo, Japan) with a Cu-Kα as the radiation source. The microscopic surface characteristics of polymers were analyzed by a contact angle measuring instrument (Dataphysics OCA40, Dataphysics, Filderstadt, Germany) and specific surface aperture analyzer (ASAP 2460 3.01, Micromeritics, Norcross, GA, USA).

The mass of CIP adsorbed on soil is calculated according to the following formula:

(1) $$q=(C_{0}-C_{e})V/m$$

where *q* (mg/g) is the adsorption capacity; *C*_0_ (mg/L) is the initial concentration of the antibiotic; *C_e_* (mg/L) is the equilibrium concentration of the antibiotic; *V* (L) is the volume of the solution; and *m* is the mass of the soil.

(2) Adsorption kinetics models

In this study, a pseudo-first-order kinetic model and a pseudo-second-order kinetic model were selected to fit the adsorption kinetic data. The pseudo-first-order kinetic model equation is shown as follows:

(2) $$ln\;\left(q_{e}-q_{t}\right)=lnq_{e}-k_{1}t$$

The pseudo-second-order kinetic model equation is shown as follows:

(3) $$\frac{t}{q_{t}}=\frac{1}{k_{2}q_{e}^{2}}+\frac{t}{q_{e}}$$

where *q_e_* (mg/g) is the amount of antibiotic at equilibrium; *q_t_* (mg/g) is the amount of antibiotic at *t* (h); *t* (h) is the adsorption time; *k*_1_ (1/h) is the pseudo-first-order kinetic adsorption reaction rate constant; and *k*_2_ (g‧h/mg) is the pseudo-second-order kinetic adsorption reaction rate constant.

(3) Adsorption models

Henry, Freundlich, and Langmuir’s adsorption models were used to fit the adsorption isotherms of CIP. Briefly, the Henry model can be described as

(4) $$q=K_{d}C_{e}\;$$

where *q* (mg/g) is the absorbed amount of antibiotic; *C_e_* (mg/L) is antibiotic mass in the aqueous phase at equilibrium the equilibrium; and *K_d_* (L/g) is the partition coefficient. The Freundlichmodel is given by

(5) $$logq=logK_{f}+1/n\;logC_{e}$$

where *K_f_* (L/g) is the Freundlich adsorption coefficient which indicates adsorption capacity and *n* is the Freundlich isotherm exponent that determines the non-linearity. The Langmuir isotherm model can be expressed as follows:

(6) $$\frac{C_{e}}{q}=\frac{1}{Q_{m}K_{L}}+\frac{C_{e}}{Q_{m}}$$

where *Q_m_* (mg/g) represents the maximum adsorption capacity and *K_L_* (L/mg) is related proportionally to the affinity between plastic particles and antibiotics.

## 3. Results and Discussion

### 3.1. Characterization of MPs

**Figure 1:**
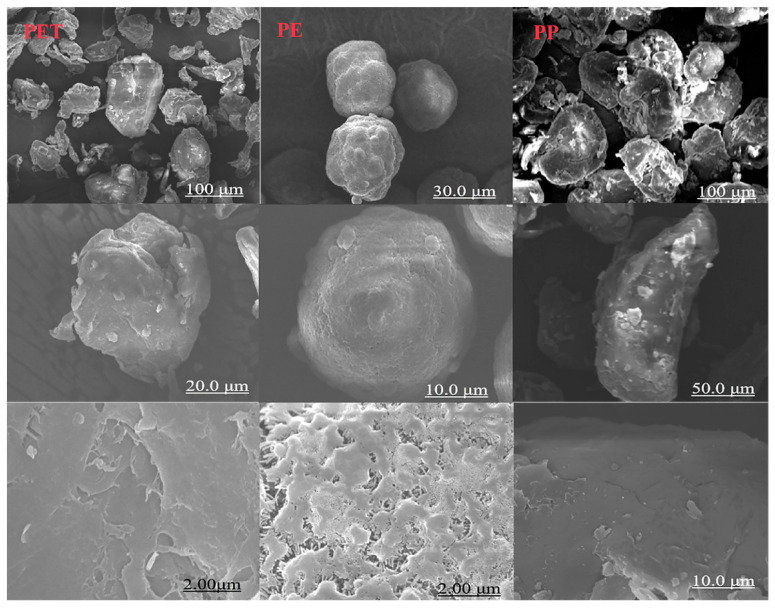
SEM micrographs of PET, PE, and PP.

SEM images of PET, PE, and PP are presented in Figure 1 (The magnifications were 10 k, 20 k, and 100 k, respectively). PET and PP particles exhibited irregular shapes with layered surface structures and relatively smooth morphology under electron microscopy. Surface protrusions observed on both polymers enhanced their specific surface area, facilitating substance adsorption. In contrast, PE displayed spherical morphology characterized by surface roughness and abundant micropores. The results that the SEM images show were similar to those of previous studies [27]. Quantitative analysis in Table S2 revealed that PP possessed the highest specific surface area, measuring 3.6 times that of PE and 2.3 times that of PET. Furthermore, PP demonstrated significantly smaller pore sizes compared to the other microplastics.

**Figure 2:**
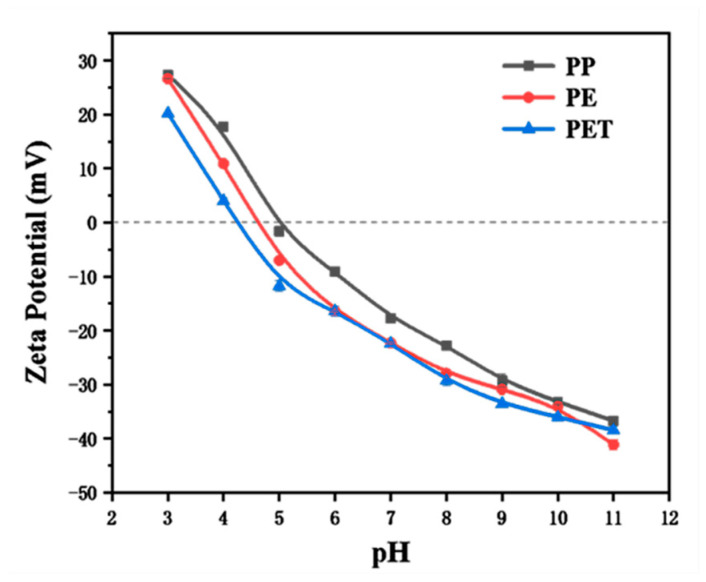
The zeta potentials of PET, PE, and PP with pH.

The zeta potential test results for PET, PE, and PP are shown in Figure 2. The zeta potentials of these MPs decreased with increasing pH, which is consistent with the conclusions of Guo et al. [28]. Among the three MPs, PP exhibited the highest zero-charge point, slightly above 5. According to electrostatic interaction principles, when the solution’s pH exceeds the zero-charge point of the MPs, they are more likely to adsorb positively charged organic molecules, followed by neutral ones, while negatively charged organic molecules would experience electrostatic repulsion.

**Figure 3:**
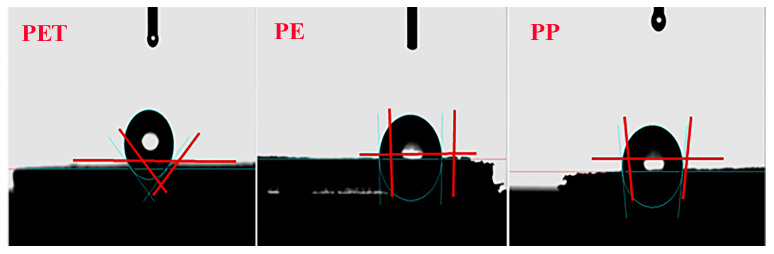
Surface water contact angle test of PET, PE and PP.

The XRD patterns are shown in Figure S1 and the results of the surface water contact angle test are shown in Figure 3 and Table S3. It shows that PET, PE, and PP had hydrophobic surfaces, and the hydrophobicity of PET was much higher than those of PE and PP.

### 3.2. Adsorption Behavior of CIP on Soil, Soil–MPs, and MPs

#### 3.2.1. Adsorption Kinetics

**Figure 4:**
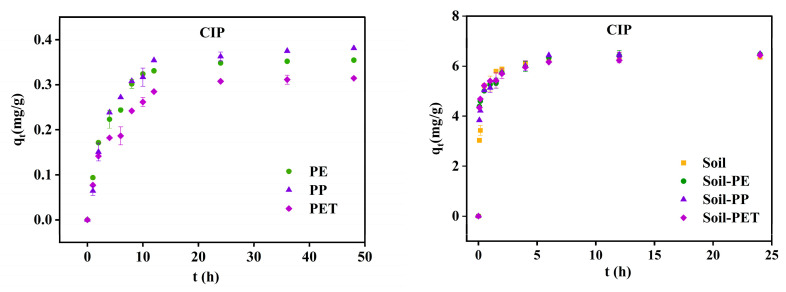
Adsorption kinetics of CIP on soil, soil–MPs, and MPs.

The adsorption kinetics of CIP onto soil, soil–MP composites, and MPs are illustrated in Figure 4. The process can be divided into three distinct phases. The first phase represents a rapid adsorption period, characterized by a high adsorption rate and a swift increase in adsorption capacity. This phase is driven by the concentration gradient-induced mass transfer and the abundance of available adsorption sites on both MPs and soil. During this stage, CIP adsorption reached 65–83% of the total adsorption capacity. The second phase corresponds to a slower adsorption period, during which the adsorption rate declined, though adsorption quantities continued to rise. The third phase marks the equilibrium stage, where equilibrium was achieved at 12 h for PE and PP, and 24 h for PET. The maximum adsorption capacities were 0.355 mg/g (PE), 0.381 mg/g (PP), and 0.314 mg/g (PET). For soil and soil–MP composites, equilibrium was reached within 6 h, with total adsorption capacities of 6.360 mg/g (soil), 6.468 mg/g (soil-PE), 6.484 mg/g (soil–PP), and 6.435 mg/g (soil–PET). Among the MPs, PP demonstrated the highest adsorption capacity due to its rubber-like nature, which facilitates rapid diffusion. Additionally, PP has a larger specific surface area and lower crystallinity compared to PE, further enhancing its ability to adsorb CIP. Overall, the adsorption rate and capacity of CIP in soil and soil–MP composites exceeded those of MPs alone. Notably, incorporating 1% MPs into soil did not significantly alter the adsorption behavior of CIP by soil.

In order to understand the adsorption process, pseudo-first-order and pseudo-second-order kinetic models were used to fit the adsorption results, which are shown in Table S4. It can be seen that the performance of the pseudo-second-order dynamic model was significantly superior to that of the pseudo-first-order model, as evidenced by *R^2^* values exceeding 0.99. This indicates that the CIP adsorption process was governed by multiple adsorption steps. The fitted parameter *q_e_* closely matched the experimental equilibrium adsorption capacity, suggesting that mechanisms such as external liquid-film diffusion, surface adsorption, and particle diffusion may influence the adsorption of CIP. Examination of the pseudo-second-order kinetic adsorption rate constant (*k*_2_) across the four soil–MP composite systems revealed that incorporating 1% MPs into the soil consistently reduced the adsorption rate of CIP, irrespective of the MP type. However, according to equilibrium adsorption capacity data in Table S4, the presence of 1% MPs had a negligible impact on the equilibrium adsorption capacity of CIP by the soil.

#### 3.2.2. Adsorption Isotherms

**Figure 5:**
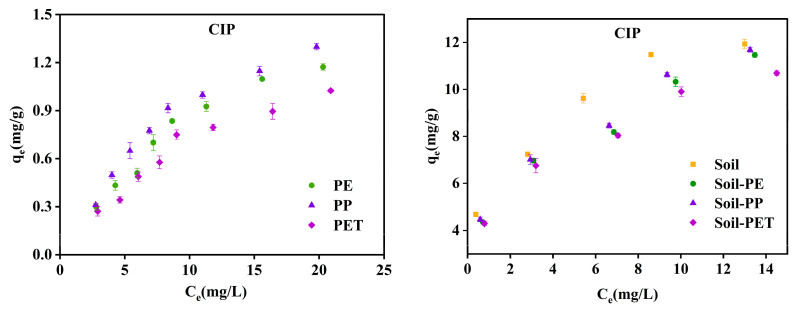
Adsorption isotherms of CIP on soil, soil–MPs, and MPs.

The Henry model, Langmuir model, and Freundlich model were used to fit the adsorption isotherm data of CIP on soil and soil–microplastic composite systems. The isothermal adsorption process is shown in Figure 5 and the fitting results are shown in Table S5. The adsorption of CIP on soil, soil–MPs, and MPs is better described by the Freundlich model, indicating that the process involves multiple adsorption forces. The Freundlich model is applicable for heterogeneous coverage and multilayer adsorption, implying that stronger binding sites are initially occupied, followed by subsequent adsorption at weaker sites. The parameter reflects the influence of concentration on adsorption capacity, where a smaller *1/n* value signifies better adsorption performance [29]. Specifically, this suggests that the adsorbent easily adsorbs CIP onto particles, while indicating difficulty in adsorption. Based on the fitting parameters in Table S5, the *1/n* value for CIP adsorption falls within the range of 0.1–1, indicating that the adsorption reaction occurs relatively easily and that the adsorption trend slows down as CIP concentration increases. Given the high value of the Henry model and the range of the Freundlich model (0.1–1), it is hypothesized that hydrophobic partitioning may play a significant role in the adsorption process. Additionally, the adsorption process aligns more closely with the nonlinear model, suggesting that it is influenced not only by partitioning effects but also by other factors, such as electrostatic interactions and hydrogen bonding.

As shown in Figure 5, the order of CIP adsorption capacity was: soil > soil–PP > soil–PE > soil–PET > PP > PE > PET. This indicates that CIP adsorption in the soil–MPs system primarily relies on soil adsorption, while the contribution of CIP adsorption by MPs can be considered negligible. Additionally, the adsorption capacity of CIP in soil decreased upon the introduction of MPs. The variation in the maximum adsorption capacity (*Q_m_*) derived from the Langmuir model supports this observation, showing a significant decrease in *Q_m_* when MPs were present. These findings align with those reported by Hüffer and Metzelder [30], who observed that incorporating PE into soil diminished the adsorption capacity for atrazine and 24-butylric acid.

### 3.3. Effect of MP Dosage on CIP Adsorption in Soil

**Figure 6:**
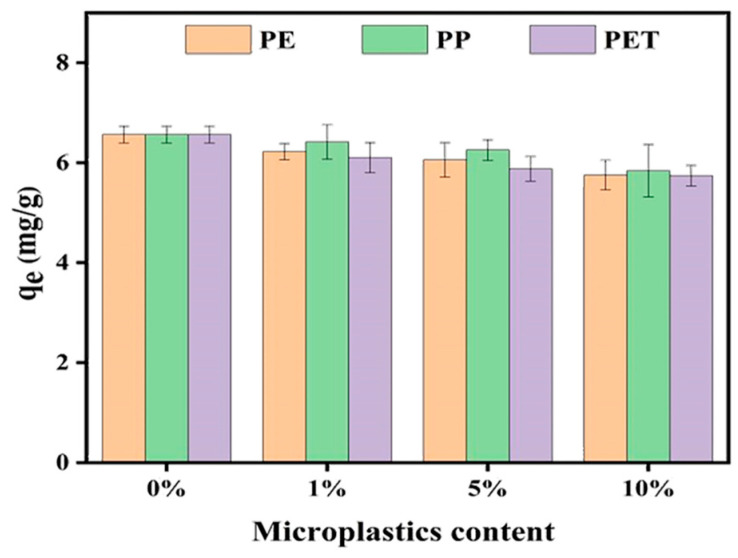
Effect of different dosages of MPs on the adsorption of CIP onto soil.

As shown in Figure 6, increasing the dosage of PE, PP, and PET MPs in soil from 0% to 10% resulted in a decrease in CIP adsorption capacity in soil, from 6.564 mg/L to 5.805 mg/L, 5.840 mg/L, and 5.741 mg/L, respectively, corresponding to reductions of 11.6%, 10.8%, and 12.5%. A gradual decline in CIP adsorption amounts on soil was observed with increasing MP dosage. This phenomenon occurred because the added MPs occupied a portion of the soil’s adsorption sites, thereby reducing the soil’s ability to adsorb CIP. These findings align with research on heavy metals [31], suggesting that the presence of MPs in soil can diminish its capacity to adsorb toxic pollutants, including antibiotics, pesticides, and heavy metals. Consequently, the free-state proportion of these pollutants increases, potentially enhancing their bioavailability. Furthermore, this could facilitate pollutant migration to deeper soil layers and elevate the risk of leaching [28].

### 3.4. Effect of pH on CIP Adsorption onto Soil and Soil–MPs

**Figure 7:**
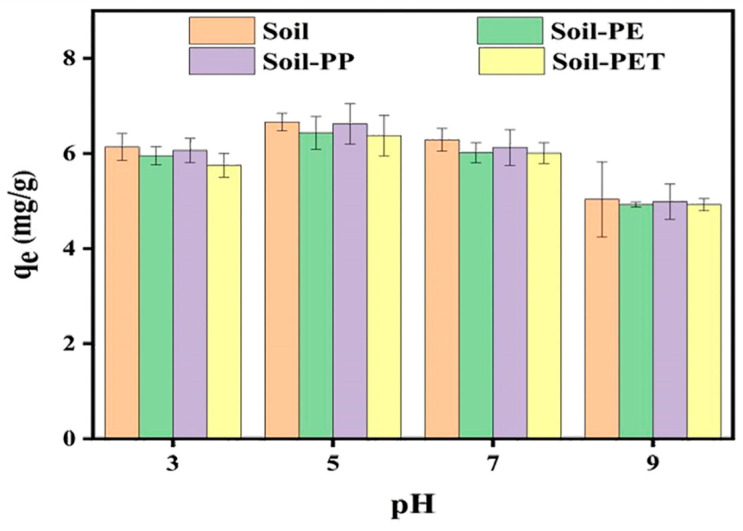
The adsorption capacity of CIP on soil and soil–MPs at different pH.

As shown in Figure 7, the CIP adsorption capacity of the soil and soil–MPs systems initially increased and then decreased with rising pH. The highest adsorption capacities occurred at pH 5, reaching 83%, 81%, 83%, and 79.9% for the four systems, respectively. Conversely, the lowest adsorption capacities were observed at pH 11, with values of 63.1%, 61.7%, 62.5%, and 61.7%, respectively. Between pH 3 and 9, the adsorption capacities of CIP in the soil, soil–PE, soil–PP, and soil–PET systems decreased by 24.4%, 23.5%, 24.8%, and 22.9%, respectively. The red soil used in this study is acidic, meaning its surface carries a negative charge. The microplastics (MPs) tested—PET, PE, and PP—had point-of-zero charge (PZC) values of pH_PZC_ = 4.3, pH_PZC_ = 4.6, and pH_PZC_ = 5.0, respectively (as shown in Figure 2). When pH > pH_PZC_, the surfaces of these MPs also become negatively charged. CIP, being an ionic organic pollutant with pKa values of 6.1 and 8.4, exists predominantly in its cationic form (CIP^+^) when pH < 6.1. Under such conditions, CIP+ readily binds to the negatively charged surfaces of both soil and MPs.

At pH = 3, the adsorption capacity of CIP was lower compared to pH = 5. This was primarily due to the high concentration of H^+^ ions competing with CIP^+^ for adsorption sites on the soil and MPs, thereby reducing CIP adsorption. Consequently, the strongly acidic environment hindered CIP adsorption. At pH = 3, the surfaces of PE, PP, and PET became positively charged, causing electrostatic repulsion with the positively charged CIP^+^. This repulsion contributed to the lower adsorption capacity of CIP in soil–MP systems compared to pure soil under the same pH condition. As pH increased, the competitive adsorption effect of H^+^ gradually diminished. At pH = 5, competition was minimized, and CIP adsorption reached its maximum. Between pH = 6.08 and pH = 8.4, CIP in solution predominantly existed as a zwitterion (CIP^±^). Beyond pH = 8.4, CIP transitioned to its anionic form (CIP⁻). In this pH range, both the soil and MP surfaces remained negatively charged, leading to electrostatic repulsion and a gradual decline in CIP adsorption capacity. This suggests that the adsorption mechanism of CIP in soil involves electrostatic interactions and cation exchange adsorption, consistent with findings reported by El-Shafey el and Al-Lawati [32].

### 3.5. Effect of Ionic Strength and Type on CIP Adsorption onto Soil and Soil–MPs

**Figure 8:**
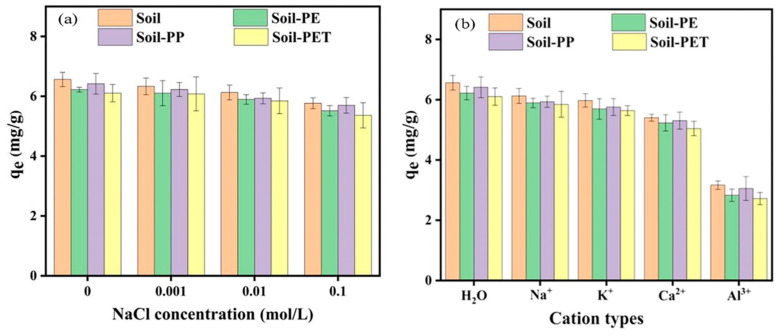
Adsorption of CIP onto soil and soil–MPs under different conditions: (**a**) NaCl solution with different concentrations; (**b**) different cation types.

Increased ionic strength reduces the adsorption capacity of CIP in soil or soil–MP systems. The addition of Na^+^, K^+^, Ca^2+^, and Al^3+^ further decreased CIP adsorption. As shown in Figure 8a, the adsorption capacity of CIP in soil or soil–MP systems progressively declined with increasing Na^+^ concentration, indicating stronger CIP adsorption under low ionic strength conditions. Under various solution conditions, the adsorption capacity followed the following order: H_2_O > Na^+^ > K^+^ > Ca^2+^ > Al^3+^ (Figure 8b). This pattern arose because Na^+^ competed with cationic CIP^+^ for adsorption sites on soil and MPs. Additionally, differences in cation valency influenced the competitive effects on active adsorption sites, altering CIP adsorption behavior in soil. Trivalent cations exhibited greater inhibitory effects than monovalent or divalent cations, consistent with the principle that higher cation valency enhances competition for negatively charged adsorption sites. While metal ions can form complexes with antibiotics via cation bridging, increasing solid-phase adsorption [33] in this study, adsorption capacity decreased with metal cation addition, suggesting limited cation bridging effects. Notably, Al^3+^ demonstrated the strongest inhibitory effect on soil CIP adsorption.

### 3.6. Adsorption Mechanism

**Figure 9:**
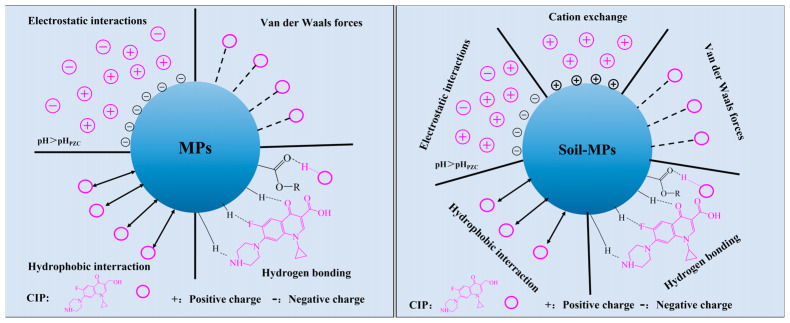
Potential adsorption mechanisms of CIP onto MPs and soil–MPs (+ and • represent positive and negative charge).

Based on the results discussed above, the adsorption capacity of CIP follows the sequence: soil > soil + MPs >> MPs. Considering the hydrophobic nature and zeta potential of MPs, hydrophobicity-driven interactions and electrostatic interactions are likely the dominant mechanisms for CIP adsorption onto MPs, alongside intermolecular forces such as van der Waals interactions. Additionally, since CIP consists of polar molecules containing nitrogen, oxygen, and fluorine atoms, and PET is derived from ester-containing polymers, hydrogen bonding may also contribute to the interaction between CIP and MPs. Soil, being a complex multi-phase system enriched with minerals, organic matter, and microorganisms, exhibits a more intricate set of adsorption mechanisms for CIP, including cation exchange, surface hydrophobic interactions, electrostatic interactions, and other processes. These complexities likely account for the significantly higher adsorption capacity of soil compared to isolated MPs. The introduction of MPs into soil alters its adsorption capacity, possibly due to the occupation of soil’s adsorption sites by the added MPs. A proposed adsorption mechanism for CIP on MPs and the soil–MP composite system is illustrated in Figure 9.

## 4. Conclusions

This study systematically examined how MPs affect ciprofloxacin (CIP) adsorption in soil systems. Results revealed similar three-stage adsorption patterns (rapid → slow → equilibrium) across soil, soil–MP mixtures, and MPs, with adsorption capacity ranking soil > soil–MPs >> MPs. Isotherm analysis suggested multi-layer adsorption governed by both chemical and physical processes. The adsorption processes were affected by the amount of added MPs, pH, ionic strength, and types. The addition of MPs weakened the adsorption capacity of soil for CIP. When pH = 5, the adsorption capacity was the highest. The adsorption capacity decreased with the increase of ionic strength, and trivalent cations had a stronger inhibitory effect on soil adsorption of CIP than monovalent and divalent cations. It was found that hydrophobic interaction and electrostatic interaction might be important mechanisms for CIP adsorption onto MPs, and cation exchange and electrostatic interaction might be important mechanisms for CIP adsorption in the soil–MPs systems.

## Data Availability

The data are contained within the article.

## Supplementary Materials

The following supporting information can be downloaded at https://www.mdpi.com/article/10.3390/toxics13040294/s1, Table S1: Basic physical and chemical properties of tested soil; Table S2: Analysis for specific surface area; Table S3: Test results of surface water contact angle of PET, PE, and PP; Table S4: Adsorption kinetics fitting parameters of CIP; Table S5: The fitting parameters of adsorption isotherms of CIP on soil, soil–MPs, and MPs; Figure S1: The XRD patterns of PET, PE, and PP.
