# Supplementary material for: Impact of Microplastics on Ciprofloxacin Adsorption Dynamics and Mechanisms in Soil
Source: Toxics. 2025 Apr 11;13(4):294. doi: 10.3390/toxics13040294 (PMC12030773; doi:10.3390/toxics13040294)
Supplement: Supplementary file 1 [file toxics-13-00294-s001.zip › toxics-3561181-supplementary.pdf]

Supplementary Materials

The basic physical and chemical properties of the red soil were showed in Table S1.

Table S1. Basic physical and chemical properties of tested soil.

Soil type	Sampling point	pH	Organic matter content（g/kg）	Cation exchange capacity（cmol/kg）
Red soil	Yingtan, Jiangxi	5.09±0.07	5.3±0.4	10.5±0.9

As showed in Table S2, the specific surface area of PP was the largest, which was 1.0818 m^2/g, about 3.6 times of PE and 2.3 times of PET. And the pore size of PP was much smaller than that of the other MPs. PET and PP had larger BJH pore volume that might be caused by agglomeration.

Table S2 Analysis for specific surface area

MPs	Specific surface area (m^2/g)	BJH pore volume (cm3/g)	Aperture (nm)
PET	0.4674	0.000853	14.6760
PE	0.3002	0.000322	12.8349
PP	1.0818	0.000745	4.5501

The XRD patterns were showed in Figure S1. All three microplastics, PET, PE, and PP, have sharp diffraction peaks, indicating that their crystallinity is relatively high. However, the intensity of the diffraction peak of PE is significantly higher than that of PP and PET. PET, PE, and PP are all semi-crystalline polymers, and their crystallinity is in the order of: PE > PP > PET.

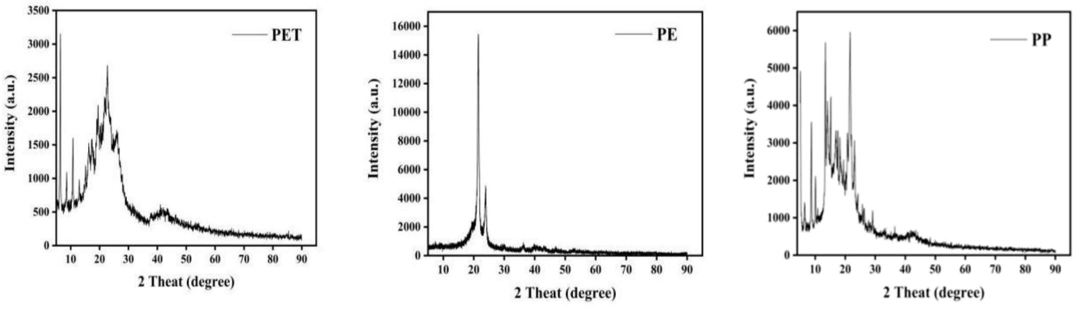

Figure S1. The XRD patterns of PET, PE and PP

The results of surface water contact angle test were showed in Table S3. It showed that PET, PE and PP had hydrophobic surfaces, and the hydrophobicity of PET was much higher than those of PE and PP.

Table S3 Test results of surface water contact angle of PET, PE and PP

Soil type	Sampling point	pH	Organic matter	Cation exchange

			content（g/kg）	capacity（cmol/kg）
PET	Left	134.8	139	135.5
	Right	134.5	139	135.2
PE	Left	87.5	95.7	92.3
	Right	87.1	96.2	91.3
PP	Left	98.1	93.6	97.7
	Right	97.6	93.2	97.3

It was found that the fitting effect of the pseudo-second-order dynamic model was significantly better than that of the pseudo-first-order dynamic model and the R^2 values reached 0.99 above (Table S4). It indicated that the CIP adsorption process was the results of multiple adsorption stages.

Table S4 Adsorption kinetics fitting parameters of CIP

**Adsorption system**	**Pseudo-first-order kinetic model**			**Pseudo-second-order dynamic model**		
	**q_e (mg/g)**	**k_1 (h)**	**R^2**	**q_e (mg/g)**	**k_2 (g/mg•h)**	**R^2**
PE	0.234	1.798	0.961	0.055	2.29	0.909
PP	0.220	1.622	0.944	0.067	2.261	0.937
PET	0.176	1.741	0.955	0.054	1.921	0.899
Soil	5.823	3.449	0.975	0.653	13.175	0.925
Soil-PE	3.968	2.793	0.966	0.419	12.837	0.937
PE	0.234	1.798	0.961	0.055	2.29	0.909

According to the fitting parameters in Table S5, the 1/n of CIP adsorption is between 0.1-1, indicating that the adsorption reaction is relatively easy to occur, and the adsorption trend gradually slows down with the increase of CIP concentration.

Table S5 The fitting parameters of adsorption isotherms of CIP on soil, soil-MPs, and MPs

**Adsorption system**	**Freundlich**			**Langmuir**			**Henry**	
	**K_f**	***n***	**R^2**	**K_L (L/mg)**	**Q_m (mg/g)**	**R^2**	**K_d (L/g)**	**R^2**
PE	0.234	1.798	0.961	0.055	2.29	0.909	0.051	0.894
PP	0.220	1.622	0.944	0.067	2.261	0.937	0.054	0.893
PET	0.176	1.741	0.955	0.054	1.921	0.899	0.041	0.893
Soil	5.823	3.449	0.975	0.653	13.175	0.925	0.578	0.905
Soil-PE	3.968	2.793	0.966	0.419	12.837	0.937	0.552	0.971
PE	0.234	1.798	0.961	0.055	2.29	0.909	0.051	0.894
